# The cAMP-HMGA1-RBP4 system: a novel biochemical pathway for modulating glucose homeostasis

**DOI:** 10.1186/1741-7007-7-24

**Published:** 2009-05-21

**Authors:** Eusebio Chiefari, Francesco Paonessa, Stefania Iiritano, Ilaria Le Pera, Dario Palmieri, Giuseppe Brunetti, Angelo Lupo, Vittorio Colantuoni, Daniela Foti, Elio Gulletta, Giovambattista De Sarro, Alfredo Fusco, Antonio Brunetti

**Affiliations:** 1 Dipartimento di Medicina Sperimentale e Clinica 'G. Salvatore', 88100 Catanzaro, Italy; 2 Cattedra di Endocrinologia, Università 'Magna Græcia' di Catanzaro, 88100 Catanzaro, Italy; 3 Dipartimento di Biologia e Patologia Cellulare e Molecolare c/o Istituto di Endocrinologia ed Oncologia Sperimentale del CNR, Università di Napoli 'Federico II', 80131 Napoli, Italy; 4 Dipartimento di Scienze Biomolecolari e Biotecnologie, Università di Milano, 20133 Milan, Italy; 5 Dipartimento di Scienze Biologiche ed Ambientali, Facoltà di Scienze MM.FF.NN., Università del Sannio, 82100 Benevento, Italy; 6 Dipartimento di Biochimica e Biotecnologie Mediche, Università di Napoli 'Federico II', 80131 Napoli, Italy

## Abstract

**Background:**

We previously showed that mice lacking the high mobility group A1 gene (*Hmga1*-knockout mice) developed a type 2-like diabetic phenotype, in which cell-surface insulin receptors were dramatically reduced (below 10% of those in the controls) in the major targets of insulin action, and glucose intolerance was associated with increased peripheral insulin sensitivity. This particular phenotype supports the existence of compensatory mechanisms of insulin resistance that promote glucose uptake and disposal in peripheral tissues by either insulin-dependent or insulin-independent mechanisms. We explored the role of these mechanisms in the regulation of glucose homeostasis by studying the *Hmga1*-knockout mouse model. Also, the hypothesis that increased insulin sensitivity in *Hmga1*-deficient mice could be related to the deficit of an insulin resistance factor is discussed.

**Results:**

We first show that HMGA1 is needed for basal and cAMP-induced retinol-binding protein 4 (*RBP4*) gene and protein expression in living cells of both human and mouse origin. Then, by employing the *Hmga1*-knockout mouse model, we provide evidence for the identification of a novel biochemical pathway involving HMGA1 and the RBP4, whose activation by the cAMP-signaling pathway may play an essential role for maintaining glucose metabolism homeostasis *in vivo*, in certain adverse metabolic conditions in which insulin action is precluded. In comparative studies of normal and mutant mice, glucagon administration caused a considerable upregulation of HMGA1 and RBP4 expression both at the mRNA and protein level in wild-type animals. Conversely, in *Hmga1*-knockout mice, basal and glucagon-mediated expression of RBP4 was severely attenuated and correlated inversely with increased *Glut4 *mRNA and protein abundance in skeletal muscle and fat, in which the activation state of the protein kinase Akt, an important downstream mediator of the metabolic effects of insulin on Glut4 translocation and carbohydrate metabolism, was simultaneously increased.

**Conclusion:**

These results indicate that HMGA1 is an important modulator of *RBP4 *gene expression *in vivo*. Further, they provide evidence for the identification of a novel biochemical pathway involving the cAMP-HMGA1-RBP4 system, whose activation may play a role in glucose homeostasis in both rodents and humans. Elucidating these mechanisms has importance for both fundamental biology and therapeutic implications.

## Background

Insulin resistance is a metabolic condition found relatively frequently among humans with chronic hyperinsulinemia and in experimental animal models with defective insulin signaling [1-3]. Recently, a link has been established between peripheral insulin sensitivity and the retinol (vitamin A) metabolism, and insulin resistance in rodents and humans has been linked to abnormalities of the vitamin A signaling pathway [4-6]. According to these studies, impaired glucose uptake in adipose tissue results in secondary systemic insulin resistance through release of the adipose-derived serum RBP4 [4,5]. However, it is unknown whether RBP4 effects on insulin sensitivity are vitamin A-dependent or vitamin A-independent. RBP4 (also called RBP) is mainly produced by the liver but also by adipocytes [7]. In plasma, retinol-RBP4 is found in an equimolar complex with transthyretin (TTR), which is a thyroid hormone transport protein that is synthesized in and secreted from the liver. This ternary complex prevents retinol-RBP4 excretion by the kidney [8]. By impairing insulin signaling in muscle, RBP4 inhibits glucose uptake and interferes with insulin-mediated suppression of glucose production in the liver, causing blood glucose levels to rise [4]. Conversely, mice lacking the *RBP4 *gene show increased insulin sensitivity, and normalizing increased RBP4 serum levels improves insulin resistance and glucose intolerance [4].

HMGA1 is a small basic protein that binds to adenine-thymine (A-T) rich regions of DNA and functions mainly as a specific cofactor for gene activation [9]. HMGA1 by itself has no intrinsic transcriptional activity; rather, it can transactivate promoters through mechanisms that facilitate the assembly and stability of a multicomponent enhancer complex, the so-called enhanceosome, that drives gene transcription [9,10].

As part of an investigation into the molecular basis regulating the human insulin receptor gene, we previously showed that HMGA1 is required for proper insulin receptor gene transcription [11,12]. More recently, we showed that loss of HMGA1 expression, induced in mice by disrupting the *HMGA1 *gene, caused a type 2-like diabetic phenotype, in which, however, impaired glucose tolerance and overt diabetes coexisted with a condition of peripheral insulin hypersensitivity [13]. Concomitant insulin resistance and insulin hypersensitivity in peripheral tissues may paradoxically coexist as observed in livers of lipodystrophic and *ob*/*ob *mice [14], as well as in *Cdk4 *knockout mice with defective pancreatic beta cell development and blunted insulin secretion [15]. The hypothesis that the paradoxical insulin hypersensitivity of *Hmga1*-deficient mice could be due to a deficit, in these animals, of RBP4 is supported by our data. Herein, by employing the *Hmga1*-knockout mouse model, we provide compelling evidence for the identification of a novel biochemical pathway involving HMGA1 and RBP4, whose activation by the cAMP pathway may play an important role in maintaining glucose metabolism homeostasis *in vivo*, in both rodents and humans. The importance of HMGA1 in *RBP4 *gene transcription was substantiated in *Hmga1*-deficient mice, in which loss of HMGA1 expression considerably decreased *RBP4 *mRNA abundance and RBP4 protein production.

## Results

### RBP4 gene transcription is induced by HMGA1 and cAMP

**Figure 1:**
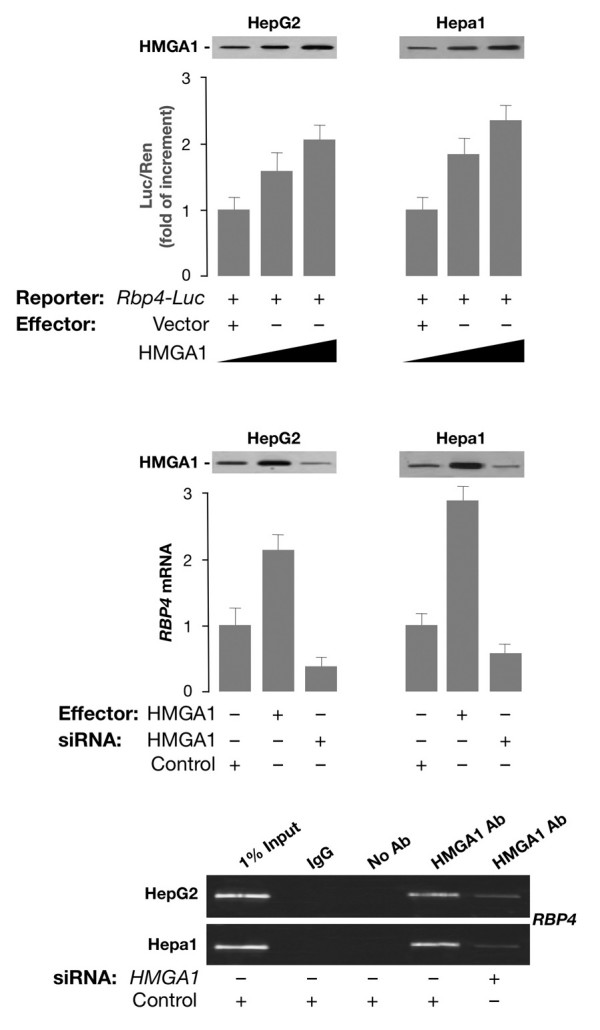
***RBP4 *gene expression is induced by HMGA1**. **(Top) **Mouse *RBP4*-Luc reporter vector (2 μg) was transfected into HepG2 and Hepa1 cells plus increasing amounts (0, 0.5, or 1 μg) of *HMGA1 *expression plasmid. Data represent the means ± standard errors for three separate experiments; values are expressed as factors by which induced activity increased above the level of Luc activity obtained in transfections with *RBP4*-Luc reporter vector plus the empty expression vector, which is assigned an arbitrary value of 1. **(Middle) ***HMGA1 *expression plasmid was transfected into HepG2 and Hepa1cells. After 6 h of transfection, the cells were treated with anti-*HMGA1 *(100 pmol), siRNA, or a non-targeting control siRNA, and endogenous *RBP4 *mRNA expression was measured 48 to 96 h later. Western blots of HMGA1 in each condition are shown in the autoradiograms. **(Bottom) **ChIP of the *RBP4 *promoter gene in HepG2 and Hepa1 cells, either untreated or pretreated with *HMGA1 *siRNA. ChIP was done using an anti-HMGA1 specific antibody (Ab).

**Figure 2:**
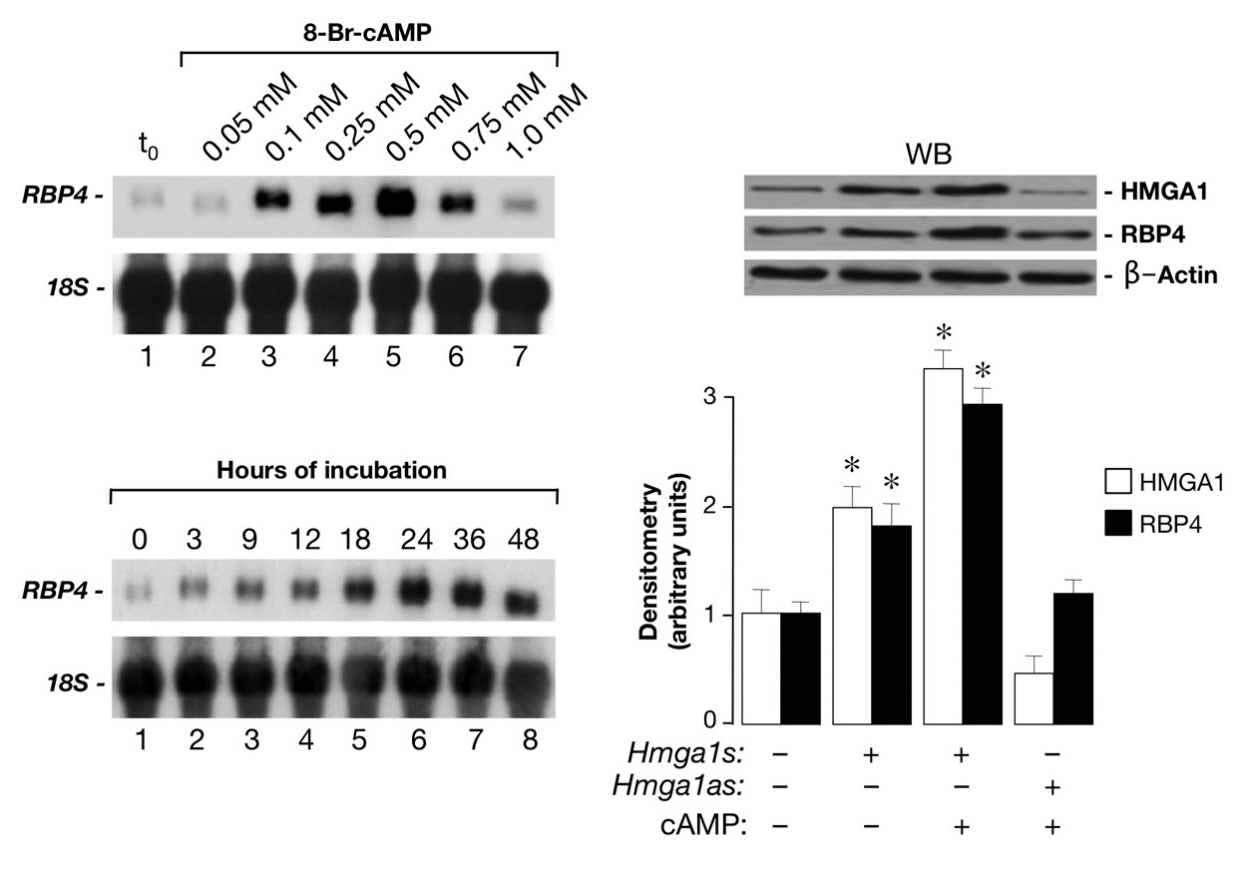
**Stimulation of *RBP4 *mRNA and protein expression by cAMP and HMGA1**. **(Upper left) **20 μg of total RNA from Hepa1 cells treated with the indicated concentrations of Br-cAMP for 24 h (lanes 1–7) were analysed by Northern blot. Hybridization was carried out with an *RBP4 *cDNA or an *18S *RNA probe as a control of the RNA loaded on each lane. **(Lower left) **20 μg of total RNA from Hepa1 cells treated with 0.5 mM Br-cAMP for the indicated times were loaded on each lane (lanes 1–8) and analysed as above. **(Right) **Hepa1 cells, in the absence or presence of an expression plasmid (1 μg) containing the *HMGA1 *cDNA in either the sense (*s*) or antisense (*as*) orientation, were left untreated or treated with Br-cAMP (0.5 mM), total protein extracts were prepared 48 h later and HMGA1 and RBP4 protein expression levels were detected by Western blot (WB) with anti-HMGA1 and anti-RBP4 antibodies, respectively. β-actin, control of cellular protein loading. Densitometric analyses of three to five independent blots are shown.

**Figure 3:**
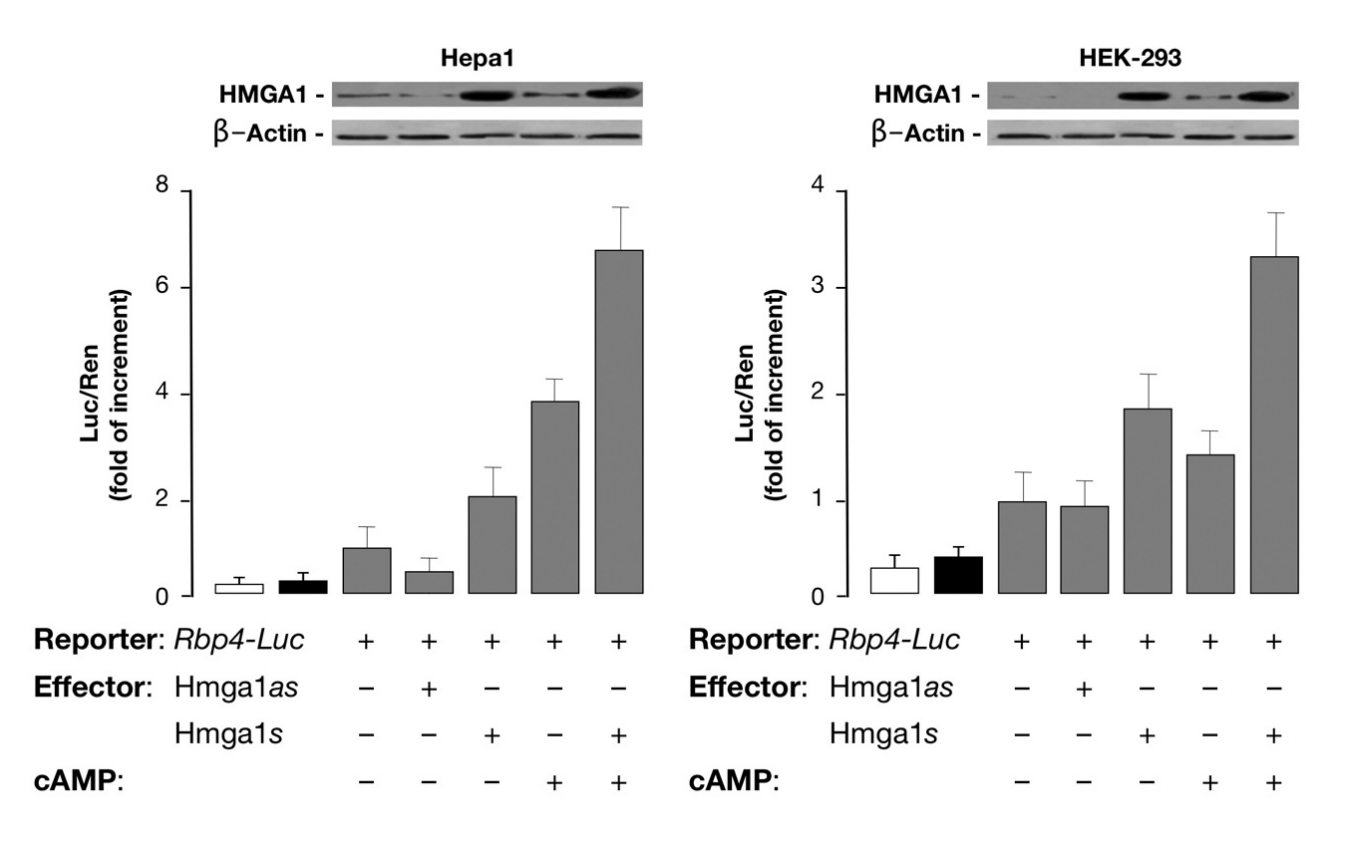
**Role of HMGA1 in basal and cAMP-induced RBP4 expression**. *Rbp4*-Luc reporter vector and *HMGA1 *expression plasmid (sense or antisense) were cotransfected into Hepa1 and HEK-293 cells, either untreated or treated with Br-cAMP. Data represent the means ± standard errors for three separate experiments. Transcriptional activity of the *RBP4 *gene promoter is shown as the ratio of luciferase activity to *Renilla *activity (Luc/Ren) as described in the experimental procedures. Values are expressed as the factors by which induced activity increased above the level of Luc activity obtained in transfections with the reporter vector alone, which is assigned an arbitrary value of 1. Open bar, mock (no DNA); black bar, pGL3-basic (vector without an insert). Western blots of HMGA1 and β-actin in each condition are shown in the autoradiograms.

We first performed experiments to see whether HMGA1 had a role in activating the mouse *RBP4 *gene promoter at the transcriptional level. To test this possibility, HepG2 human hepatoma cells and mouse Hepa1 hepatoma cells were cotransfected transiently with mouse *RBP4*-Luc reporter plasmid plus increasing amounts of the HMGA1 expression vector. As shown in Figure 1, overexpression of HMGA1 considerably increased *RBP4*-Luc activity in both cell types and this effect occurred in a dose-dependent manner. Consistent with these results, *RBP4 *mRNA abundance was increased in cells overexpressing HMGA1 and was reduced in cells pretreated with siRNA targeting HMGA1 (Figure 1), indicating that activation of the *RBP4 *gene requires HMGA1. These data were substantiated by chromatin immunoprecipitation (ChIP) assay, showing that binding of HMGA1 to the endogenous *RBP4 *locus was increased in whole, intact HepG2 and Hepa1 cells naturally expressing HMGA1, and was decreased in cells exposed to siRNA against HMGA1 (Figure 1). Based on these results, in addition to previous observations indicating that cAMP, or agents which elevate intracellular cAMP, increase *RBP *transcript levels [16], we were interested to see whether a functional link could be established between cAMP, HMGA1, and RBP4. To this end, we first confirmed and extended the observation made by Jessen and Satre [16] that *RBP *is induced by cAMP in Hepa1 cells. As measured by Northern blot analysis of total RNA (Figure 2), *RBP4 *mRNA increased ≈ 5-fold over the basal level in Hepa1 cells treated with 0.5 mM 8-bromo cAMP (Br-cAMP), a standard concentration for c-AMP induction experiments [16]. As shown in Figure 2, *RBP4 *mRNA levels increased starting at 3 h, peaking at 24 h and then declining, suggesting a transient transcriptional stimulation. To establish whether HMGA1 was required for basal and cAMP-dependent *RBP4 *transcription, we transfected the HMGA1 expression vector in Hepa1 cells treated or not with Br-cAMP and RBP4 protein levels were analyzed 48 h later by Western blot. As shown in Figure 2, RBP4 protein expression was enhanced in cells overexpressing HMGA1 and even further in cells treated with cAMP, in which an increase in HMGA1 protein expression was simultaneously observed, suggesting that induction of RBP4 by cAMP may occur, at least in part, through activation of endogenous HMGA1 expression. This hypothesis was supported by the fact that RBP4 was reduced in cAMP treated cells in which endogenous levels of HMGA1 were specifically lowered by transfecting cells with HMGA1 antisense expression plasmid (Figure 2). However, further experiments are needed to fully explain the role of cAMP on HMGA1 expression. The functional significance of HMGA1 in *RBP4 *gene expression was confirmed in transient transcription assays in Hepa1 (and differentiated 3T3-L1, data not shown) cells, in which overexpression of HMGA1 caused an increase in both basal and cAMP-induced Luc activity from the mouse *RBP*-Luc reporter plasmid (Figure 3). This effect was substantiated in HEK-293 cAMP-responsive cells, a cell line ideally suited for studying the effects of HMGA1 on transcription since it does not express appreciable levels of this protein. As shown in Figure 3, in support of the role that HMGA1 plays in the context of *RBP4 *gene, the direct effect of cAMP was less effective in promoting *RBP4 *transcription in HEK-293 cells expressing low levels of HMGA1, becoming considerably higher in cells with forced expression of HMGA1.

Thus, these data together demonstrate that HMGA1 is of major importance for transcriptional regulation of the *RBP4 *gene, and indicate that a functional link exists between cAMP, HMGA1, and RBP4.

### Hmga1-deficient mice have reduced expression of RBP4 in liver and fat tissue and reduced serum RBP4 levels

**Figure 4:**
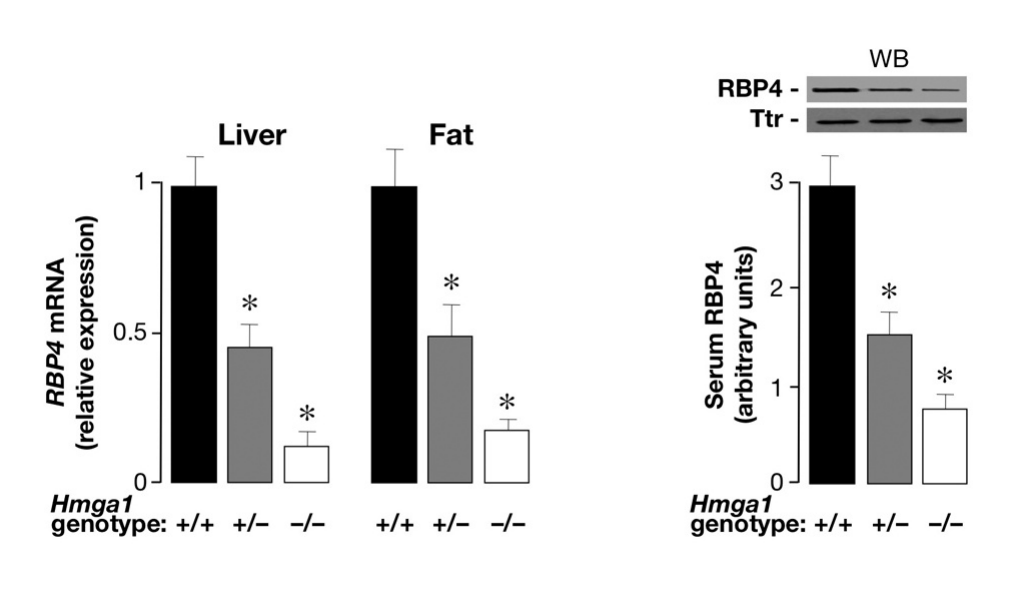
**RBP4 expression in wild-type and *Hmga1*-deficient mice**. RBP4 mRNA in liver and fat from control and *Hmga1*-deficient mice, as measured by qRT-PCR (left), and densitometric quantification of RBP4 serum levels as detected by Western blot (WB) of serum samples (2 μl) from mice with diverse genotypes (right). In WB analysis, an anti-transthyretin (TTR) antibody was used to confirm similar amounts of protein on each lane. Results are from 4–6 mice in each group. *P < 0.01 versus control mice.

In the light of the above experimental results, indicating that HMGA1 plays a positive role in *RBP4 *gene transcription in living cultured cells, it was interesting to analyze the functional consequences of genetic ablation of HMGA1 on RBP4 *in vivo*, in *Hmga1*-knockout mice. To this end, we performed studies aimed at investigating the expression of *RBP4 *mRNA and protein in *Hmga1*-deficient mice and wild-type controls. As shown in Figure 4, *RBP4 *mRNA was severely attenuated in both liver and fat from *Hmga1*-null mice, and reduced by 50% in *Hmga1 *heterozygous mutants, as assessed by real-time quantitative polymerase chain reaction (qRT-PCR). Reduced *RBP4 *mRNA levels in liver and adipose tissue paralleled the decrease in RBP4 serum levels as detected by Western blot analysis of serum samples from age- and body weight-matched mice with diverse genotypes (Figure 4), thereby showing the requirement of HMGA1 for full RBP4 expression in whole animals.

### HMGA1 and RBP4 expression increase in liver and fat of normal mice after intraperitoneal glucagon injection

**Figure 5:**
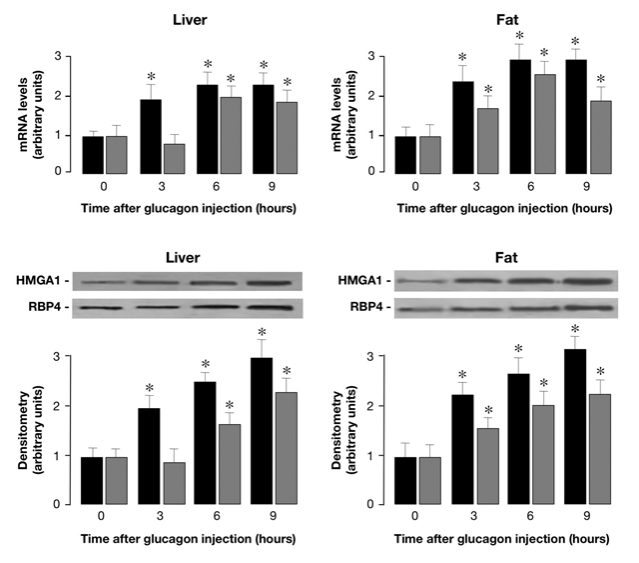
***Hmga1 *and *RBP4 *mRNA and protein expression *in vivo*, in glucagon-injected wild-type mice**. Total RNA was isolated from liver (upper left) and fat (upper right) of 3-h-fasted mice, before and after intraperitoneal injection of glucagon. Levels of *Hmga1 *and *RBP4 *mRNA were measured at the indicated time intervals by qRT-PCR and normalized to *RPS9 *mRNA abundance, as described in *Methods*. Results are the mean values ± s.e.m. from 4–6 animals per group. Black bars, *Hmga1 *mRNA; gray bars, *RBP4 *mRNA. Representative Western blots from liver (lower left) and fat (lower right) of mice before and after glucagon injection are shown. Densitometric analyses of immunoblots are shown in bar graphs as the mean ± s.e.m. of data from 3–5 mice per each time point. Black bars, HMGA1; gray bars, RBP4.*P < 0.05 versus control mice (time 0).

**Figure 6:**
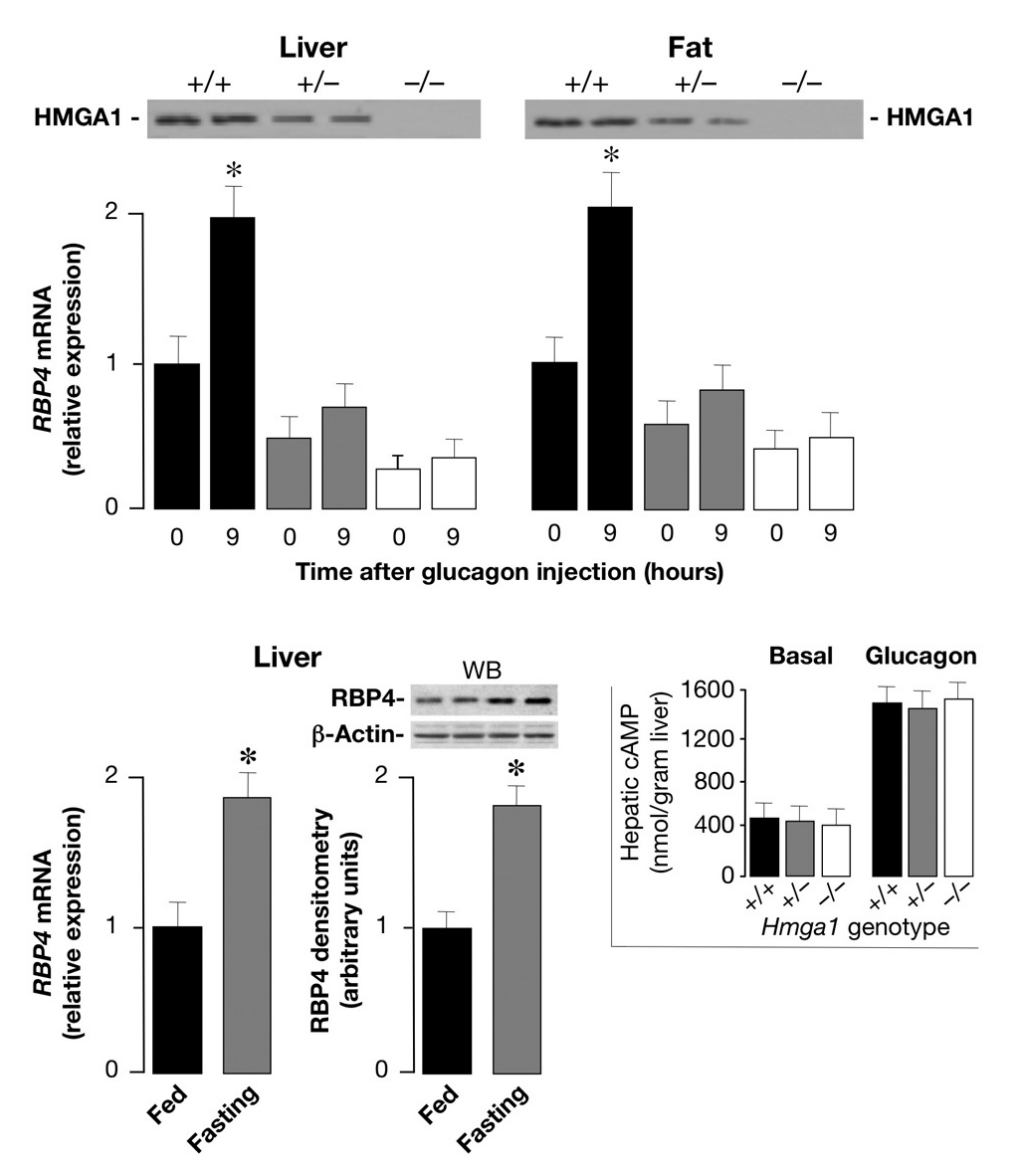
**Comparison of *RBP4 *mRNA levels in glucagon-injected wild-type and *Hmga1*-deficient mice, and liver RBP4 expression in wild-type mice during fasting and fed**. Total RNA was isolated from liver and fat of 3-h-fasted mice, before (time 0) and after 9 h of intraperitoneal injection of glucagon, and *RBP4 *mRNA was measured by qRT-PCR and normalized to *RPS9 *mRNA abundance. Results are the mean values ± s.e.m. from 6–8 animals per group. Black bars, *Hmga1*^+/+^, *n *= 8; gray bars, *Hmga1*^+/-^, *n *= 6; white bars, *Hmga1*^-/-^, *n *= 6. *P < 0.05 versus each control (time 0). Western blots for HMGA1 protein expression are shown in liver and fat from all three genotypes (top). The levels of *RBP4 *mRNA and protein (shown at the bottom of the figure) were measured in liver of fed and 6-h-fasted wild-type mice (6 animals per group), using qRT-PCR and Western blot (WB), respectively. *P < 0.05 versus fed mice. Inset, cAMP was measured in liver from control and *Hmga1*-deficient mice, in both basal conditions and 3 h after the intraperitoneal injection of glucagon (1 mg/kg body weight), as described in the *Methods *section. The data are mean ± s.e.m. for 4–6 animals per group.

Based on our observations in intact cultured cells, indicating a role for the cAMP signaling pathway in *HMGA1 *and *RBP4 *gene expression, cAMP-inducible transcriptional activation of the *Hmga1 *and *RBP4 *genes was investigated *in vivo*, in whole animals, by systemic administration of the intracellular cAMP-elevating hormone glucagon. Under these conditions, glucagon-stimulated cAMP responses in terms of both *Hmga1 *and *RBP4 *mRNA expression were first analyzed in wild-type control mice. Consistent with our data in Hepa1 cells, *Hmga1 *and *RBP4 *mRNA levels significantly increased in liver and fat of normal mice after intraperitoneal injection of glucagon (Figure 5). Time course analyses revealed that the induction and accumulation of *Hmga1 *mRNA preceded the expression of *RBP4 *mRNA in both tissues. In liver, *RBP4 *mRNA appeared after that for *Hmga1*, peaked after 6 h following glucagon injection, and then remained at a plateau (Figure 5). In fat, *RBP4 *mRNA appeared at 1 h after *Hmga1 *mRNA, peaked after 6 h of glucagon stimulation, and decreased smoothly thereafter (Figure 5). Increased levels of *Hmga1 *and *RBP4 *mRNAs were paralleled by the increase of Hmga1 and RBP4 protein expression, as measured by Western blot analysis of proteins from liver and fat of glucagon-injected animals (Figure 5). Interestingly, when similar experiments were carried out in glucagon injected *Hmga1*-deficient mice, tissue expression of *RBP4 *mRNA was severely attenuated in liver and fat from heterozygous (*Hmga1*^+/-^) and *Hmga1*-null (*Hmga1*^-/-^) animals (Figure 6), thereby indicating that HMGA1 is indeed required for maximal induction of the *RBP4 *gene *in vivo*, in the whole organism, and that the glucagon/adenylate cyclase system regulates both *HMGA1 *and *RBP4 *gene and protein expression. Consistent with this conclusion, liver *RBP4 *mRNA and protein expression levels were lower in fed wild-type mice, becoming higher during fasting, when circulating glucagon increases (Figure 6).

As a measure of the glucagon efficacy in glucagon-injected mice, a liver biopsy was taken before and after glucagon injection, and cAMP levels in liver were determined for both control and *Hmga1*-deficient mice (Figure 6, inset). No substantial difference was found in basal levels of cAMP (0.45 and 0.50 in *Hmga1*^-/- ^and *Hmga1*^+/-^, respectively, versus 0.52 μmol/g tissue in controls). After glucagon injection, hepatic levels of cAMP increased to 1.50 μmol/g tissue in control mice, compared with 1.48 and 1.52 in *Hmga1*^-/- ^and *Hmga1*^+/- ^mice, respectively. Results similar to those shown in the inset of Figure 6 were also obtained in epididymal and subcutaneous fat pads from control and mutant animals (data not shown), thus indicating that the glucagon-stimulated cAMP synthesis did not differ among mice with diverse genotypes.

### Hmga1-deficient mice have increased Glut4 expression and insulin signaling activity in skeletal muscle and fat

**Figure 7:**
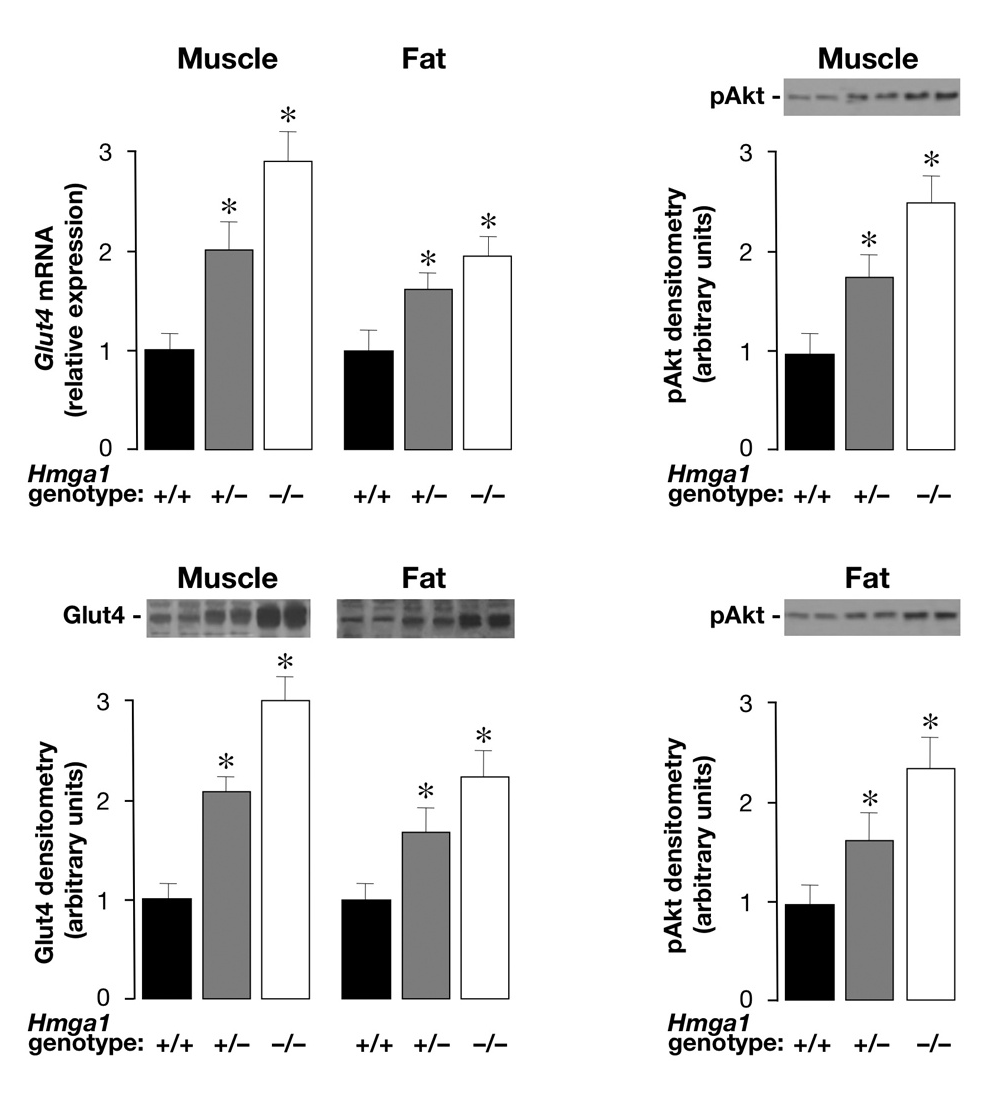
**Glut4 and pAkt expression in wild-type and *Hmga1*-deficient mice**. *Glut4 *mRNA (upper left) and protein content (lower left) in muscle and fat parallel pAkt protein abundance in skeletal muscle (upper right) and adipose tissue (lower right) from control and *Hmga1*-deficient mice. Representative Western blots of Glut4 and pAkt proteins are shown, together with the densitometric analyses of six to eight independent blots. Black bars, *Hmga1*^+/+^, *n *= 8; gray bars, *Hmga1*^+/-^, *n *= 6; white bars, *Hmga1*^-/-^, *n *= 6. *P < 0.05 versus *Hmga1*^+/+^.

Systemic insulin resistance has been associated with elevation of serum RBP4, whereas genetic and pharmacological interventions aimed at decreasing serum RBP4 levels enhance insulin action and improve insulin sensitivity [4]. Increased peripheral insulin sensitivity during insulin-tolerance test was previously observed by us in *Hmga1*-knockout mice [13]. To verify whether a functional link indeed existed between HMGA1 and RBP4, and whether insulin hypersensitivity in *Hmga1*-deficient mice could be mediated by the HMGA1-RBP4 system, we carried out quantitative measurements of *Glut4 *mRNA transcript abundance. Examination by qRT-PCR showed a significant increase of *Glut4 *transcripts in both skeletal muscle and adipose tissues from *Hmga1*-deficient mice compared with controls (Figure 7). Accordingly, immunoblotting of muscle and fat tissue showed a 2- to 3-fold increase of Glut4 in the insulin hypersensitive *Hmga1*-knockout mice compared with controls (Figure 7), clearly indicating that an inverse correlation between RBP4 and Glut4 indeed exists *in vivo*, in this animal model of diabetes, in which reduced RBP4 may contribute to the maintenance of glucose homeostasis by increasing insulin signaling and peripheral insulin sensitivity. In agreement with this interpretation, the activation state of the protein kinase Akt, an important downstream target of PI 3-kinase regulating insulin serum effects on Glut4 translocation and carbohydrate metabolism [17], was increased in mutant animals. As shown in Figure 7, basal phospho-Akt immunoreactivity was higher in skeletal muscle and adipose tissues from *Hmga1*-deficient mice compared with wild-type controls, and this increase paralleled closely the increase of Glut4 protein in adipose and muscle plasma membranes from heterozygous and homozygous *Hmga1 *mutants. In line with previous observations on transcriptional repression of the mouse *Glut4 *gene by cAMP [18], endocrine upregulation of Glut4 in *Hmga1*-deficient mice was substantiated further by *in vitro *experiments (not shown), indicating that in isolated adipocytes treated with Br-cAMP, *Glut4 *mRNA was decreased in all three genotypes. A positive correlation of RBP4 levels with markers of lipid metabolism adversely affecting insulin sensitivity has been reported recently in both clinical and experimental studies [19,20]. *Hmga1*-knockout mice had lower levels of serum free fatty acids (0.45 ± 0.13 and 0.34 ± 0.07 in *Hmga1*^+/+ ^and *Hmga1*^-/-^, respectively; *P *< 0.05), which might contribute to their improved insulin sensitivity.

### Recombinant RBP4 injection reduces Glut4 and insulin signaling activity in muscle and fat tissue of Hmga1-deficient mice, and attenuates insulin hypersensitivity of these animals

**Figure 8:**
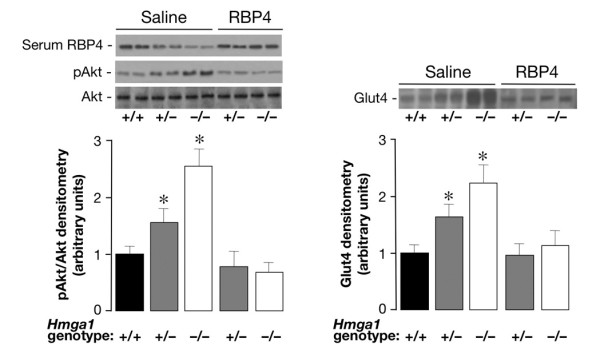
**Effects of recombinant RBP4 administration on Akt phosphorylation and Glut4 protein expression in *Hmga1*-deficient mice**. Basal (saline) levels of pAkt (left) and Glut4 (right) were increased in skeletal muscle of saline-injected *Hmga1*-deficient mice compared with controls, and were reduced following RBP4-injection (*n *= 6 per genotype). Densitometric quantifications of three independent experiments from 3 animals per genotype are shown, together with representative Western blots of pAkt, Glut4, and serum RBP4 of saline and RBP4-injected mice. *P < 0.05 versus *Hmga1*^+/+^.

To demonstrate that increased insulin sensitivity in mutant mice was directly due to HMGA1 regulation of RBP4, we determined the effect of recombinant RBP4 administration on Akt phosphorylation and Glut4 protein expression in skeletal muscle from *Hmga1*-deficient mice. As shown in Figure 8, Akt phosphorylation was reduced in muscle from RBP4-injected mutant animals compared with saline-injected *Hmga1 *mutants. The reduction in Akt phosphorylation in these genotypes correlated inversely with RBP4 serum levels in the same animals (Figure 8) and paralleled the reduction of Glut4 in skeletal muscle and adipose (not shown) plasma membranes (Figure 8), indicating that, in these conditions, activation of the Akt-Glut4 pathway is regulated, at least in part, by circulating RBP4. Plasma insulin levels were slightly higher in RBP4-injected mice, but no significant difference was found (1.6 ± 0.2 and 1.2 ± 0.2 in RBP4-injected *Hmga1*^+/- ^and *Hmga1*^-/- ^respectively, versus 1.4 ± 0.1 and 0.9 ± 0.1 ng/ml in saline-injected *Hmga1*^+/- ^and *Hmga1*^-/- ^mice).

**Figure 9:**
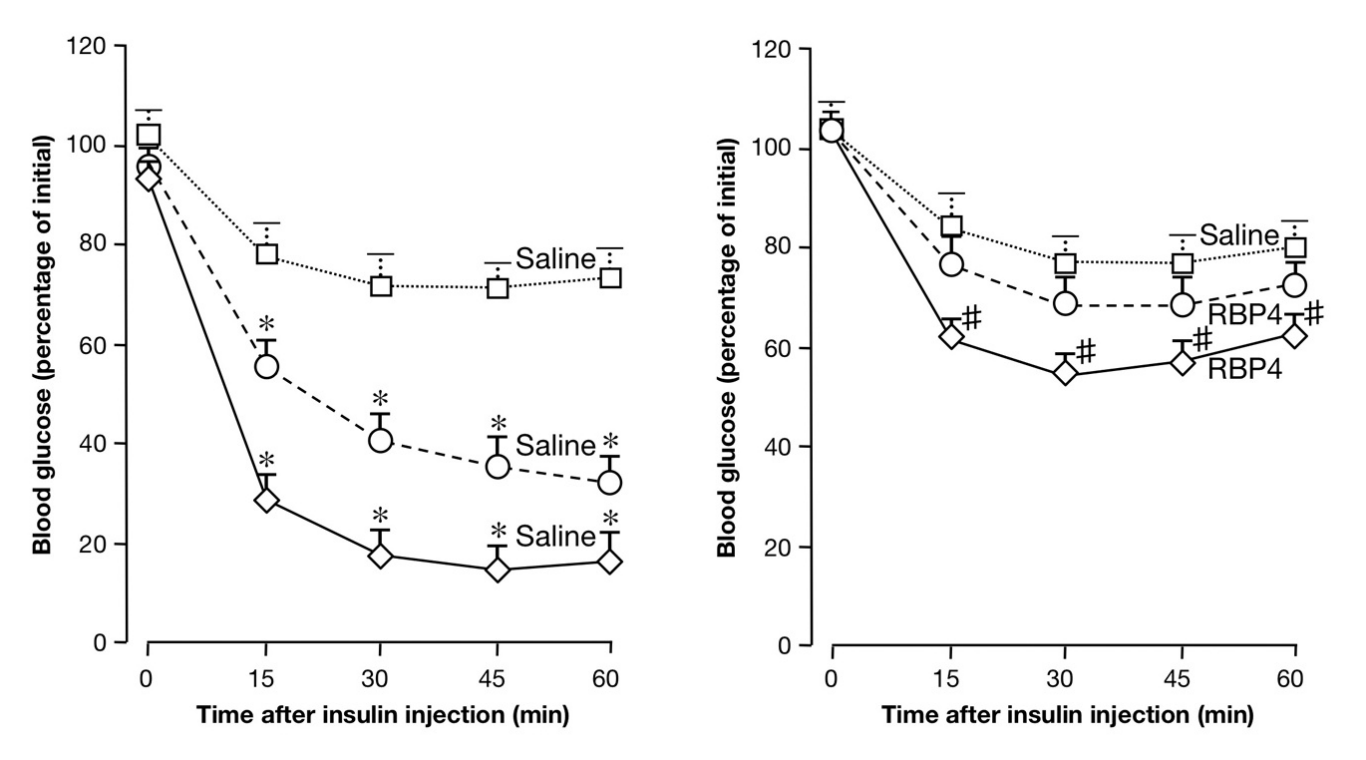
**Effects of RBP4 on insulin sensitivity**. Insulin-tolerance test (ITT) was assessed in *Hmga1*-deficient mice injected with saline alone (left), and in *Hmga1 *mutants injected chronically with purified RBP4 (right) (*n *= 6–8 per genotype in each condition). ITT was performed by measuring blood glucose levels in 12-h-fasted conscious mice injected intraperitoneally with human insulin (Human Actrapid, Novo Nordisk), 1 U/kg body weight. Open squares, *Hmga1*^+/+^; open circles, *Hmga1*^+/-^; open diamonds, *Hmga1*^-/-^. The degree of statistical significance was less in RBP4-injected *Hmga1*-deficient mice compared with the significance for saline-injected *Hmga1 *mutants. *P < 0.0001, saline-injected *Hmga1*-deficient mice versus *Hmga1*^+/+^; *# P *< 0.05, RBP4-injected *Hmga1*^-/- ^mice versus *Hmga1*^+/+^.

Consistent with the condition of insulin hypersensitivity, we previously reported that the glucose-lowering effect of exogenous insulin was enhanced in *Hmga1*-deficient mice during insulin-tolerance test (ITT) [13]. To support further the role of RBP4 in insulin hypersensitivity in *Hmga1 *mutants, we have determined the effect of RBP4 administration on the glucose fall induced by insulin in these genotypes during ITT. As shown in Figure 9, injection of human RBP4 in heterozygous and homozygous *Hmga1 *mutants caused a less dramatic fall in blood glucose levels, lessening the hypoglycemic response to intraperitoneal insulin observed in the saline-injected animals. Thus, taken together, our findings consistently support the role of HMGA1 as a key element in the transcriptional regulation of genes involved in glucose metabolism and add new insights into the compensatory mechanisms that may contribute to counteract insulin resistance *in vivo*. By directly regulating *RBP4 *gene transcription, HMGA1 enhances peripheral insulin sensitivity, ensuring glucose uptake in skeletal muscle. This, if on one hand might represent an adaptive mechanism to ameliorate insulin resistance in animals with a disadvantageous metabolic risk profile, on the other might indicate that the cAMP/HMGA1-mediated RBP4 expression during fasting (when glucagon peaks) may act physiologically to reduce insulin sensitivity in peripheral tissues, thereby contributing to the maintenance of euglycemia under this condition. This was supported by the observation that after an overnight fasting period (12–16 h) plasma glucose concentration in wild-type mice was higher than that of *Hmga1*-deficient mice (89 ± 5 in *Hmga1*^+/+ ^mice, versus 72 ± 6 and 62 ± 5 mg/dl in *Hmga1*^+/- ^and *Hmga1*^-/- ^mice, respectively; *P *< 0.05).

## Discussion

We have previously shown that loss of HMGA1 protein expression, induced in mice by disrupting the *HMGA1 *gene, severely decreased insulin receptor expression (below 10% of control animals) and phosphorylation in the major targets of insulin action, largely impaired insulin signaling, and reduced insulin secretion, producing a type 2-like diabetic phenotype in which defects in both peripheral insulin sensitivity and pancreatic beta-cell insulin secretion were coexpressed simultaneously [13]. However, despite the severe decrease in insulin receptor signaling and insulin receptor production, the glucose-lowering effect of exogenous insulin was enhanced in *Hmga1*-deficient mice during ITT, and the glucose infusion rate necessary to maintain euglycemia was higher in mutant mice during hyperinsulinemic-euglycemic clamp [13], supporting the existence of alternative pathways of insulin signaling promoting glucose uptake and disposal in certain adverse metabolic conditions such as those found in the *Hmga1*-knockout mouse. The existence of signaling pathways promoting glucose uptake and utilization in peripheral tissues through mechanisms that are independent of insulin has been postulated before, on the basis of experimental observations supporting the existence of molecular circuits/pathways that can compensate for the decrease in insulin-stimulated glucose uptake *in vivo*, in both animal models and human patients with type 2 diabetes [21-23]. However, how these compensatory mechanisms are activated has remained hitherto largely undefined. As previously shown, consistent with the ubiquitous distribution of HMGA1, insulin receptor expression was also reduced in pancreatic tissue from *Hmga1*-deficient mice [13]. Loss of insulin secretion in response to glucose has been reported in IRβ knockout mice with tissue-specific knockout of the insulin receptor in pancreatic beta cells [24]. As in the IRβ knockout mice, plasma insulin after glucose challenge was considerably reduced in *Hmga1*-mutant animals, in which the acute first-phase insulin secretory response was severely blunted [13], indicating a glucose-induced insulin secretory defect. In addition, substantial abnormalities in pancreatic islet morphology and size have been described in *Hmga1*-knockout mice [13], indicating that decreased insulin secretion in this genotype may also depend on reduced beta-cell mass. Thus, defects in both pancreatic beta-cell insulin secretion and peripheral insulin action coexist simultaneously in this knockout mouse model of diabetes, in which activation of compensatory mechanisms to efficiently overcome these metabolic abnormalities may be of vital importance.

Downregulation of Glut4 in adipose tissue is a typical feature of insulin-resistant states, such as obesity and type 2 diabetes [25]. It has been found that the decrease in Glut4 expression that occurs in the fatty tissue of obese animals and humans is accompanied by increased expression and secretion of the adipocyte-derived RBP4 fraction [4,5], suggesting that RBP4 production is tightly regulated by adipose tissue glucose uptake. RBP4 has been recently implicated in systemic insulin sensitivity in rodents and humans, in which elevated serum RBP4 levels were associated with reduced expression of Glut4 in adipocytes, and correlated inversely with peripheral insulin sensitivity. However, based on current data, the role of RBP4 in insulin sensitivity in humans is still controversial and might be restricted to rodent models only. Interspecies differences are known to exist and discrepancies between humans and mice might emphasize the role of non-genetic environmental factors and genetic modifiers in determining the phenotypic variations in RBP4 and insulin sensitivity between humans and animal models. Our results in the present study clearly indicate that in *Hmga1*-knockout mice RBP4 levels are considerably decreased in serum and in whole liver and adipose tissue extracts, strictly linking HMGA1 and RBP4 expression. We propose that HMGA1 deficiency adversely affects RBP4 expression and this, in animals with a disadvantageous metabolic risk profile like that observed in the *Hmga1*-knockout mouse model, might reflect an adaptive mechanism to increase glucose uptake and glucose disposal. Consistent with the results obtained in *Hmga1*-deficient mice, RBP4 was considerably reduced in cells of both human (HepG2) and mouse (Hepa1) origin readily expressing RBP4, following perturbation of endogenous HMGA1 protein expression in cells treated with siRNA against HMGA1. Conversely, an increase in *RBP4 *mRNA abundance was observed in both cell lines following forced expression of HMGA1, consistently supporting a role for HMGA1 in the transcriptional activation of the *RBP4 *gene. These findings were substantiated further by ChIP analysis, showing that HMGA1 indeed binds to the *RBP4 *locus in intact living cells.

Signal transduction pathways which raise intracellular cAMP have been reported to have a potential role in the regulation of *RBP4 *gene expression [16]. Although the molecular mechanisms underlying this effect remain poorly understood, evidence exists supporting the notion that the regulation of *RBP4 *gene transcription via the cAMP signaling pathway may be physiologically relevant. One important physiological condition in which intracellular cAMP increases is in response to low glucose availability. In this metabolic setting, a concomitant predominance of circulating counter-regulatory hormones, in particular pancreatic glucagon acting via the cAMP pathway, induces glycogenolysis and gluconeogenesis in the liver, which produce and release hepatic glucose in the blood. In this regard, the cAMP-element-binding protein (CREB) has been identified as a critical transcriptional checkpoint which, in response to cAMP, promotes hepatic glucose output through the synergistic activation of distinct transcriptional effector pathways, which include the PPAR gamma coactivator 1 (PGC1) and the NR4A orphan nuclear receptors [26].

In this paper, we report that systemic injection of glucagon to wild-type control mice caused an increase in *RBP4 *mRNA and protein expression, along with an increase of both intracellular cAMP and HMGA1 levels. Glucagon effects were attenuated in *Hmga1*-deficient mice, supporting a distinct role for HMGA1 in the regulation of *RBP4 *gene expression and functionally linking this two genes. As a consequence of the functional link between HMGA1 and RBP4, a significant increase in *Glut4 *mRNA and protein was observed in both skeletal muscle and adipose tissues from *Hmga1*-deficient mice compared with controls. An inverse relationship between RBP4 and Glut4 has been described previously, in the adipose-*Glut4*^-/- ^mouse, in which the decrease in Glut4 expression that occurs in the fatty tissue of this mutant genotype is accompanied by increased expression and secretion of the fat-derived RBP4 [4]. In our model, instead, *RBP4 *expression is genetically impaired due to the lack of HMGA1 and Glut4 is increased in both muscle and fat, suggesting that abnormalities in RBP4 and/or metabolites of the vitamin A metabolism may directly affect whole-body insulin action and peripheral insulin sensitivity. In support of this possibility, identification of regulatory single nucleotide polymorphisms in the *RBP4 *gene associated with type 2 diabetes has been recently reported [27,28], while correlations of RBP4 with insulin resistance have been confirmed in experimental clinical approaches in humans [7]. Although conflicting results have been reported, raising doubt about the postulated relationship of RBP4 with insulin sensitivity in humans, our results in *Hmga1*-deficient mice confirm that an inverse correlation indeed exists between RBP4 and insulin sensitivity *in vivo*, in this animal model of diabetes, lending support to previous hypotheses that lowering RBP4 levels would be helpful in ameliorating insulin resistance, at least in mice.

Overall, our findings provide mechanistic insight into the regulation of glucose uptake and disposal in peripheral tissues, and support further the role of HMGA1 as a molecule that is likely to be an important emerging factor in the transcriptional activity of genes implicated in the maintenance of glucose homeostasis and metabolic control, such as the insulin receptor gene [11-13], the leptin gene [29], and, as shown here, the *RBP4 *gene. Apart from the intrinsic biological interest in elucidating the mechanisms leading to improvement in insulin sensitivity, a clear understanding of the molecular process involved is of potential importance in the development of new therapeutic strategies for patients with metabolic disorders such as obesity, diabetes, and other insulin resistant states.

## Conclusion

We propose that HMGA1 can serve as a modulator of both *RBP4 *gene expression and protein function and represents an important novel mediator of glucose homeostasis *in vivo*.

## Methods

### Plasmids, transfections, and ChIP

The *RBP4*-Luc reporter plasmid was obtained by cloning the *NheI*/*XhoI *1427-bp sequence of the mouse *RBP4 *promoter (-1417 to +10) into pGL3 (Promega). This fragment was amplified from genomic DNA using the following modified primers: 5'-TTGCTAGCATGGCTAAGGTGCTTGTTGAAA-3', 5'-TTCTCGAGCACACCCACTCCATCTCACC-3' and the integrity of this construct was checked by DNA sequencing. *RBP4*-Luc reporter plasmid, together with either the control vector plasmid or expression plasmid encoding HMGA1 [11], was transiently transfected into cultured cells using LipofectAMINE 2000 reagent (Invitrogen), and Luc activity was assayed 48 h later, as previously described [30]. Renilla control vector served as an internal control of transfection efficiency, together with measurements of protein expression levels. For antisense HMGA1 experiments, *RBP4*-containing vector was cotransfected into Hepa1 cells with the expression plasmid pcDNA1 containing the *HMGA1 *cDNA in the antisense orientation [12]. Small interfering RNA (siRNA) targeted to *HMGA1 *[30] was transfected into cells at 50% to 60% confluency and cells were analyzed 48 to 96 h later. ChIP assay was performed in HepG2 and Hepa1 cells, either untreated or pretreated with *HMGA1 *siRNA as described previously [31]. Formaldehyde-fixed DNA-protein complex was immunoprecipitated with anti-HMGA1 antibody. Primers for the *RBP4 *sequence were used for PCR amplification of immunoprecipitated DNA (30 cycles), using PCR ready-to-go beads (Amersham Pharmacia Biotech). PCR products were electrophoretically resolved on 1.5% agarose gel and visualized by ethidium bromide staining.

### Animals

Male *Hmga1*-deficient and wild-type mice aged 6–9 months were studied. The generation of these animals and many of the physiological characteristics of the mice have been described in detail [13]. All animal work was carried out at the Animal Facility at the 'Istituto dei Tumori di Napoli', and at the Faculty of Pharmacy, Roccelletta di Borgia, Catanzaro, using approved animal protocols and in accordance with institutional guidelines. Serum free fatty acid levels were measured in wild-type and *Hmga1*-knockout mice (*n *= 12–16 per genotype) using the NEFA C kit (Wako).

### Real-time PCR and Western blot

For qRT-PCR, total cellular RNA was extracted from tissues using the RNAqueous-4PCR kit and subjected to DNase treatment (Ambion). RNA levels were normalized against 18S ribosomal RNA in each sample, and cDNAs were synthesized from 2 μg of total RNA using the RETROscript first strand synthesis kit (Ambion). Primers for mouse *HMGA1 *(NM_016660.2) (5'-GCAGGAAAAGGATGGGACTG-3'; 5'-AGCAGGGCTTCCAGTCCCAG-3'), *RBP4 *(NM_011255.2) (5'-AGGAGAACTTCGACAAGGCT-3'; 5'-TTCCCAGTTGCTCAGAAGAC-3'), *Glut4 *(NM_009204) (5'-TCATTGTCGGCATGGGTTT-3'; 5'-CGGCAAATAGAAGGAAGACGTA-3'), and *RPS9 *(NM_029767.2) (5'-CTGGACGAGGGCAAGATGAAGC-3'; 5'-TGACGTTGGCGGATGAGCACA-3') were designed according to sequences from the GenBank database. A real-time thermocycler (Eppendorf Mastercycler ep realplex ES) was used to perform quantitative PCR. In a 20-μl final volume, 0.5 μl of the cDNA solution was mixed with SYBR Green RealMasterMix (Eppendorf), and 0.3 μM each of sense and antisense primers. The mixture was used as a template for the amplification by the following protocol: a denaturing step at 95°C for 2 min, then an amplification and quantification program repeated for 45 cycles of 95°C for 15 s, 55°C for 25 s, and 68°C for 25 s, followed by the melting curve step. SYBR Green fluorescence was measured, and relative quantification was made against the *RPS9 *cDNA used as an internal standard. All PCR reactions were done in triplicate.

Western blot analysis was performed to analyze HMGA1 and RBP4 protein expression in whole-cell liver and fat extracts from normal and mutant mice, using polyclonal specific antibodies raised against HMGA1 [11] and RBP4 (AdipoGen, Inc.). For the measurement of serum RBP4, blood was collected from the retro-orbital sinus, plasma protein extracts were resolved on 12% SDS-PAGE, blotted onto nitrocellulose membranes and RBP4 was detected using rabbit polyclonal antisera at 1:2000 dilution, as suggested by the manufacturer. TTR was detected using a goat anti-TTR polyclonal antibody (Santa Cruz Biotechnology). Rabbit anti-Glut4 polyclonal antibody was used as previously described [13].

### In vivo studies with the peptide hormone glucagon

For systemic administration of exogenous glucagon, mice were injected in the peritoneal cavity with human glucagon (1 mg/kg body weight) or saline after 3 h of fasting. At this dose, the peak increase of plasma glucagon in all genotypes was ~96% ± 10% above pre-injection levels, reflecting similar previous observations in rodents [32]. At different times after the injection the mice were killed by cervical dislocation, the liver and fat were rapidly removed, frozen into liquid nitrogen and stored at -80°C until processed. For cAMP determination, frozen samples were first homogenized in ice-cold trichloroacetic acid (TCA) (6% wt/vol), and cAMP was determined using the cAMP enzyme immunoassay kit (Amersham Pharmacia Biotech), according to the instructions specified by the manufacturer.

### RBP4 purification and injection

Human *RBP4 *cDNA cloned into a pET3a expression vector was a kind gift from JW Kelly (The Scripps Research Institute). Based on previously published methodology [33], *RBP4 *protein expression vector was transformed into the BL21 strain of *Escherichia coli *(Stratagene), expanded in suspension culture and induced for 6 h with 1 mM isopropyl-D-thiogalactopyranoside to stimulate protein expression. Bacteria were pelleted and lysed by osmotic shock [34]. From this point on, all steps, including denaturation, refolding, and RBP4 purification, were performed essentially as described elsewhere [35]. Protein fractions were examined by sodium dodecyl sulfate polyacrylamide gel electrophoresis (SDS-PAGE) and immunoblotting, and desired fractions were pooled together, concentrated with an Amicon Centriprep-10 concentrator (Millipore), and stored at -80°C.

To determine whether elevation of RBP4 affected insulin hypersensitivity *in vivo*, in *Hmga1*-deficient mice, heterozygous and homozygous *Hmga1 *mutants, were intraperitoneally injected twice daily (at 12-h intervals) with 200 μg of purified human RBP4 (13 μg/g body weight per mouse) for 7 days. This resulted in a daily average serum level of human RBP4 similar to that of control mice (see Figure 8), which received physiological saline solution according to the same schedule above.

### Statistical analysis

The ANOVA test was used to evaluate the differences between the groups of mice. For all analyses, *P *< 0.05 was considered significant.

## Abbreviations

Akt: protein kinase B; Br-cAMP: 8-bromo cAMP; cAMP: cyclic adenosine monophosphate; CREB: cAMP-element-binding protein; Glut4: glucose transporter-4; HEK-293: human embryonic kidney-293; Hepa1: mouse hepatoma; HMGA1: high mobility group A1; ITT: insulin-tolerance test; PGC1: PPAR gamma coactivator 1; qRT-PCR: quantitative Real-Time PCR; RBP4: retinol-binding protein 4; siRNA: small interfering RNA; TCA: trichloroacetic acid; TTR: transthyretin.

## Authors' contributions

EC and FP performed qRT-PCR studies as well as transient transfections with reporter and expression vectors, and participated in the design of the study. SI was involved in Western blotting studies and assisted FP in performing transfections with siRNA. ILP participated in Western blotting and performed cloning studies. DP, EG, GDS and AF participated in the analysis and discussion of the *in vivo *data from normal and mutant mice. GB, AL, and VC performed certain aspects of the assays detailed in Figures 1, 2 and 3 and contributed with Northern blotting studies. DF provided helpful discussion on this manuscript and participated in ChIP analysis. AB conceived, coordinated, and supervised the project, analyzed data, and wrote the manuscript. All authors read and approved the final manuscript.
